# Tracing the radiation of *Maniola* (Nymphalidae) butterflies: new insights from phylogeography hint at one single incompletely differentiated species complex

**DOI:** 10.1002/ece3.1338

**Published:** 2014-12-04

**Authors:** Angelina J Kreuzinger, Konrad Fiedler, Harald Letsch, Andrea Grill

**Affiliations:** Department of Botany and Biodiversity Research, University of ViennaVienna, Austria

**Keywords:** Biogeography, DNA barcoding, endemism, expansion routes, phylogeny, speciation, species delimitation

## Abstract

The use of DNA sequence data often leads to the recognition of cryptic species within putatively well-known taxa. The opposite case, detecting less diversity than originally described, has, however, far more rarely been documented. *Maniola jurtina*, the Meadow Brown butterfly, occurs all over Europe, whereas all other six species in the genus *Maniola* are restricted to the Mediterranean area. Among them, three are island endemics on Sardinia, Cyprus, and Chios, respectively. *Maniola* species are almost indistinguishable morphologically, and hybridization seems to occur occasionally. To clarify species boundaries and diversification history of the genus, we reconstructed the phylogeography and phylogeny of all seven species within *Maniola* analyzing 138 individuals from across its range using mitochondrial and nuclear genetic markers. Examination of variation in mitochondrial and nuclear DNA surprisingly revealed a case of taxonomic “oversplitting”. The topology of the recovered phylogenetic tree is not consistent with accepted taxonomy, but rather reveals haplotype clades that are incongruent with nominal species boundaries: instead of seven species, we recognized only two major, yet incompletely segregated, lineages. Our results are consistent with the hypothesis that *Maniola* originated in Africa. We suggest that one lineage dispersed over the Strait of Gibraltar and the Iberian Peninsula to the west of Europe, while the other lineage spreads eastward through Asia Minor and over the Bosporus to Eastern Europe.

## Introduction

Taxonomic mishaps such as “oversplitting” (the misinterpretation of individual variants as distinct specific entities) and “lumping” (erroneously grouping several species into a single one) continue to pose problems in modern taxonomy and systematics (Dayrat 2005). In the last decade, DNA barcoding has become a handy tool for resolving species identifications, especially in clades where morphological characterization of putative taxa is weak or inconsistent (Dupuis et al. 2012; Puillandre et al. 2012; Ratnasingham and Hebert 2013).

As a consequence, supposedly well-established taxonomic systems for many groups of organisms have become subject to frequent, and often drastic, change due to ongoing revisions. Newly described cryptic species and novel insights into relationships between taxa frequently overturn traditional systematics, even in comparatively well-known groups such as butterflies (e.g., Dinca˘ et al. 2011; Talavera et al. 2013). In particular, the use of DNA barcoding approaches has led to a very substantial increase of recognized species numbers during the last decade (Hebert et al. 2004; Hausmann et al. 2011).

Meadow Brown butterflies have been in the focus of studies in evolutionary genetics and ecology since decades (Scali 1971; Brakefield 1982a,b; Goulson 1993). The center of these studies has been the widespread *M. jurtina*. The other species in the genus, especially the island endemics, have been largely neglected since their description, apart from a few anecdotal taxonomic papers (e.g., Jutzeler et al. 1997), some work on ecological aspects of differentiation (Grill et al. 2006a,b) and work on morphometrics in *M. jurtina* and *M. nurag*, particularly in genitalia (Dapporto 2010). *Maniola jurtina* alone has been studied more extensively, but so far, no conclusive picture of phylogeographic patterns has emerged (Schmitt et al. 2005; Dapporto et al. 2011). As taxonomic uncertainties last due to great morphological variation and overlap in distribution areas, we here investigated species boundaries with molecular markers.

*Maniola* comprises seven described species with a rather peculiar distribution: *Maniola jurtina* (Linnaeus, 1758) is widely distributed over much of Europe, northwest Africa, the Canary Islands, and eastward to the Ural mountains and NW Iran (Tshikolovets 2011), whereas the other six species in the genus are restricted to the Mediterranean area (Grill et al. 2006a, 2007). Among them, four species are narrowly endemic to islands and therefore belong to those butterflies of Europe with the smallest ranges: *M. cypricola* Graves, 1928 on Cyprus, *M. chia* Thomson, 1987 on the Aegean islands Chios and Inousses, *M. halicarnassus* Thomson, 1990 on the Aegean island Nisyros and the neighboring Turkish Bodrum peninsula, and *M. nurag* Ghiliani, 1852 on Sardinia (Grill et al. 2007; Kudrna et al. 2011). The remaining two species inhabit largely overlapping parts of the eastern Mediterranean realm: *M. telmessia* (Zeller, 1847) is found on many Aegean islands and throughout southern Turkey from the Bosporus eastward to Syria, Lebanon, Israel, Jordan, Iraq, and NW Iran (Hesselbarth et al. 1995; Tshikolovets 2011). The range of *M. megala* (Oberthür, 1909) is smaller, from the Aegean island Lesbos through southern Turkey as far as the Syrian border (Tshikolovets 2011).

With these unusual distributions, *Maniola* stands as an example for a genus with one widespread and several regional species, a pattern that is found similarly in a range of Palaearctic animal genera, not only in butterflies but in fact in many groups including vertebrates (e.g., in wall lizards *Podarcis*: Poulakakis et al. 2005; in birds of the genus *Oenanthe*: Randler et al. 2012; in butterflies: Dennis et al., 2008; Dapporto and Strumia 2008).

*Maniola jurtina*,*M. telmessia*,*M. halicarnassus,* and *M. megala* are broadly sympatric in Turkey, yet evidence for differential habitat preferences is vague (Hesselbarth et al. 1995). Despite minor variation in emergence times, hybridization has been recorded, for example between *M. halicarnassus* and *M. telmessia* (Hesselbarth et al. 1995) as well as *M. jurtina* and *M. nurag* on Sardinia (Grill et al. 2006b). All *Maniola* species strikingly resemble each other in terms of wing patterns and genitalia morphology, making taxonomy a challenging task even for experts (Thomson 1973; Olivier 1993; Grill et al. 2004). In Table1, a survey of described morphological and ecological differences of the *Maniola* species is given.

**Table 1:** Survey of morphological and ecological characters of *Maniola* species, collated from Hesselbarth et al. (1995), Olivier (1993), Tshikolovets (2011), Tolman and Lewington (2008) and Makris and John (2003)

	*M. jurtina*	*M. nurag*	*M. chia*	*M. megala*	*M. telmessia*	*M. halicarnassus*	*M. cypricola*
Wingspan	Male: 36–44 mm; female: 37–46 mm	Male: 36–40 mm, smaller than *M. jurtina*; female: 36-40 mm	Male: 36–44 mm; female: 37–46 mm	Male: 36–44 mm; female: 37–46 mm, larger than *M. jurtina*	Male: 36–45 mm, smaller than *M. jurtina*; female: 37–46 mm	Male: 42–47 mm; female: 42–47 mm, larger than *M. telmessia*	40–50 mm
Elevation	Up to 2700 m in Caucasus	500–1500 m	Up to 800 m	Up to 1000 m	Up to 2000 m	Up to 450 m	Up to 1900 m
Flight period	May–September	May–late September	May–October	May–October	April–October	May–October	April–November
Habitat	Grassy, bushy, often flowery places	Grassy, flowery places among bushes and rocks	Grassy, rocky and bushy places; cultivated ground	Similar to *M. telmessia*; grassy areas including woody and cultivated areas	Light woodlands, rocky scrublands	Moist and shaded grassy places	Everywhere on Cyprus
Ovum	11–21 longitudinal ribs, regionally variable		13–14 longitudinal ribs	19–21 longitudinal ribs	14–16 longitudinal ribs, very small eggs	9–18 longitudinal ribs	
Morphological differences		Yellow–orange areas more extensive than in *M. jurtina*; better developed, more sharply defined in females; conspicuous sex-brand in males	Indistinguishable from *M. jurtina*; distinct genitalia and differences in allozyme genetics	Hind wing outer margin averagely more undulate; underside markings darker; very short penis and spiny excrescences on basal part of gnathos	Apical ocellus always distinct and bigger than in *M. jurtina*, yellow orange encircled	Male sex-brand large, triangular; male genitalia distinguishable from *M. megala*,*M. chia* and *M. jurtina*; females more orange than all other *Maniola*	Male forewings larger, more elongated and hairy than in *M. telmessia*, triangular sex-brand; female genitalia different from *M. telmessia*
No. larval instars	6				5	Variable, 5 or 6	
Notes	Morphologically very variable species			Lesbos: smaller fore wings, highest portion of individuals with bipupillary ocelli	Emergence 2-4 weeks earlier than *M. jurtina*	Emergence 2.5 weeks after *M. telmessia*, flies at cooler, shadier places than *M. telmessia*	Larvae feed only on silicate poor grasses

Available studies on phylogeography within *Maniola* conducted so far never included all species of the genus and never had a geographically large enough taxon sampling (Schmitt et al. 2005; Grill et al. 2006a; Dapporto et al. 2011). So, this study aims at (1) reconstructing the phylogeny of the genus *Maniola*, (2) comparing it with the currently accepted taxonomy, (3) testing the usability of DNA barcoding for species identifications in *Maniola*, and (4) investigating whether molecular data reveal information about the existence of refugia in the Mediterranean region and possible expansion routes that have led to the current distribution of the species. What we find for *Maniola* may indicate that similar outcomes are to be expected for a number of other genera with similarly peculiar distribution patterns.

## Material and Methods

### Sample collection, DNA extraction, amplification, sequencing, and alignment

We used 138 *Maniola* individuals, from sites across Europe, Anatolia, and northern Africa (Table S2) representing all seven taxonomically described species. Specimens had either been dried or put into 99% ethanol after collection. Samples were then stored at −20°C until DNA extraction. The applied mitochondrial and nuclear genetic markers (cytochrome-c-oxidase [COI], cytochrome-B [CytB], wingless [wgl], and elongation factor 1*α* [EF1a]) were amplified with varying success (Table2), as the specimens had been collected between the years 1980 and 2013; older samples performed often worse in PCR than the more recent ones. We additionally included COI sequences from 51 individuals from NCBI GenBank.

**Table 2:** Successfully amplified marker sequences (mtDNA = COI + CytB; combined = all genetic markers used)

	*COI*	*CytB*	*wingless*	*EF 1α*	mtDNA	Combined
*M. jurtina* (*n* = 51)	47	29	30	28	27	22
*M. nurag* (*n* = 25)	19	12	12	11	8	9
*M. chia* (*n* = 18)	16	7	14	17	6	6
*M. megala* (*n* = 6)	5	5	5	3	5	3
*M. cypricola* (*n* = 14)	12	14	11	9	12	9
*M. telmessia* (*n* = 16)	11	16	14	13	11	11
*M. halicarnassus* (*n* = 8)	8	8	5	5	8	5
Total	118	91	91	86	77	65

As outgroups, we downloaded sequences of closely related satyrine butterfly species from GenBank (see Table S1), from the subtribes Coenonymphina, Erebiina, Maniolina, Melanargiina, Pronophilina, and Satyrina, to improve support of the phylogeny of the genus. Outgroups were selected from Peña et al. (2006), but as they did not use *CytB* in their study, we additionally sequenced two specimens of *Pyronia cecilia* that were at hand in our laboratory to have at least one species available with complete gene sampling.

Total genomic DNA was extracted from two legs, respectively, or, if missing, from thoracic muscles following a standard protocol (DNeasy Blood & Tissue Kit, Qiagen Inc., Valencia, CA). DNA samples were extracted and amplified in a separate room exclusively dedicated to DNA extractions. Primer names, references, primer sequences as well as respective annealing temperature and time are shown in Table3.

**Table 3:** Primer sequences used in this study (F = forward, R = reverse)

Gene	Primer name	References	Sequence (5′-3′)	Annealing temp. and time
*COI*	*LepF*	Hajibabaei et al. (2006)	ATTCAACCAATCATAAAGATATTGG (F)	44°C – 1 min 30 s; 46°C – 1 min 15 s
	*LepR*		TAAACTTCTGGATGTCCAAAAAATCA (R)	
*CytB*	*CB-J-10933*	Simons et al. (1994)	TATGTACTACCATGAGGACAAATATC (F)	46°C – 1 min 20 s
	*CB-N-11367*		ATTACACCT CCTAATTTATTAGGAAT (R)	
*wgl*	*LepWG1*	Brower and DeSalle (1998)	GARTAYAARTGYCAYGGYATGTCTGG (F)	48°C – 1 min 30 s
	*LepWG2*		ACTICGCARCACCARTGGAATGTRCA (R)	
*Ef 1α*	*EF51.9*	Monteiro and Pierce (2001)	CARGACGTATACAAAATCGG (F)	52.5°C – 1 min 30 s
	*EFrcM4*		ACAGCVACKGTYTGYCTCATRTC (R)	
	*Starsky/M3*	Cho et al. (1995)	CACATYAACATTGTCGTSATYGG (F)	54°C – 1 min
	*Luke/rcM51-1*		CATRTTGTCKCCGTGCCAKCC (R)	

Gene fragments of the mitochondrial (*COI*,*CytB*) and nuclear DNA (*wgl*,*EF1α*) were amplified using polymerase chain reaction (PCR) in a thermal cycler (Eppendorf Mastercycler pro S vapo.protect). Negative (sterile water) and positive (samples with known genotypes) controls were always used. PCRs were performed in 25 *μ*L volumes containing 1 *μ*L of genomic DNA, 22.5 *μ*L of ReddyMix®, 0.5 *μ*L of the respective forward and reverse primer, and 1 *μ*L BSA. PCR conditions were optimized for each primer pair.

PCR products were visualized on an agarose gel to verify amplification success. Afterward, they were analyzed using an ABI capillary sequencer (3730 DNA analyzer, Applied Biosystems). Sequences contained no gaps or stop codons and were aligned and edited in BioEdit 7.1.3.0 (Hall 1999) for each gene separately. All new sequences have been submitted to GenBank, under accession numbers KP032366 - KP032458 (Cytb), KP032241 - KP032365 (COI), KP032552 - KP032639 (EF1a), and KP032459 - KP032551 (wgl). Accession numbers of outgroup species are provided in Table S1.

### Phylogenetic analysis

We used Bayesian inference (BI) to reconstruct phylogenetic trees. The combined genes tree (including 65 individuals, for which all genetic markers worked) was based on an alignment of 2427 base pairs. Trees of COI included 118 sequences with 657 bp length. We used the program JModelTest (v. 2.0) on the Phylemon-Server 2.0 (Sánchez et al. 2011) to estimate models of nucleotide substitutions (of three substitution schemes: JC, HKY, and GTR) as judged by the Bayesian information criterion (BIC) for BI. Only the best-fit models were subsequently used for evaluations of tree topology. BI analysis was performed with MrBayes v.3.2, considering the estimated best-fit substitution models (Huelsenbeck and Ronquist 2001). Trees were visualized using Figtree v.1.3.1 (Rambaut 2009).

For BI trees under BIC, the best-fit models were as follows: [GTR + G] for *COI*, [HKY + I + G] for *CytB*, [GTR + I + G] for mtDNA and all genes combined, [K80 + I] for *Elongation factor* and nDNA, and [HKY + I] for *wingless*.

Genetic variation within described species was estimated as the numbers of variable sites (*S*), average numbers of nucleotide differences (*k*), haplotype diversity (*h*: Nei, M. 1987), and nucleotide diversity (*π*: Nei and Li 1979) for each gene as well as species, using the software DnaSP v5 (Librado and Rozas 2009). Genetic distances (Kimura-2-distances) were calculated with MEGA 5.1 (Tamura et al. 2011).

### Haplotype networks

Additionally, we generated median-joining networks for several datasets using the program Network 4.6.1.1 (http://www.fluxus-engineering.com). The median-joining method is based on a maximum parsimony algorithm that searches for all shortest trees of a particular dataset (Bandelt et al. 1999).

## Results

### Genetic diversity and species delimitation by DNA barcoding

For *COI* (657 bp), 118 sequences showed 37 different haplotypes with 48 variable sites. The 91 *CytB* sequences (432 bp) displayed 33 haplotypes and 43 variable sites. The 87 sequences of *Elongation factor* (1051 bp) showed 53 haplotypes and 66 variable sites, and *wingless* (403 bp; 91 sequences) showed 34 haplotypes and 25 variable sites.

For all species and all genetic markers (Table4), estimates of haplotype diversity (*h*) were rather high, ranging from 0.333 to 0.996, whereas estimates of nucleotide diversity (*π*) were much lower (especially in nuclear genes), ranging from 0.00112 to 0.02804. Interestingly, the endemic species did not show lower haplotype and nucleotide diversity than their mainland congeners, and *M. nurag* even showed the highest diversity of all as well as very high average numbers of nucleotide differences (*k*). The available *M. megala* specimens all had exactly the same haplotype (see Fig. 4), and *M. halicarnassus* revealed rather low numbers of variable sites. As two lineages could be detected in the phylogenetic trees (see subsection *Phylogeny and phylogeography*), they were also investigated for their nucleotide diversity: Lineage A (comprising the taxa *M. cypricola*,*M. telmessia,* and *M. halicarnassus*) generally had lower nucleotide diversity than Lineage B (all other nominal species).

**Table 4:** Summary of molecular diversity indices of *COI*,*CytB*,*wgl*,*elongation factor 1α* genes, mitochondrial DNA (combined *COI* and *CytB*), and nuclear DNA (nDNA); sample size (*n*), number of haplotypes (no.), number of variable sites (*S*), average number of nucleotide differences (*k*), haplotype diversity (*h*), and nucleotide diversity (*π*) with standard deviation (SD). Nuclear genes have been doubled for analysis by the program DNAsp to avoid ambiguous sites, as DNAsp cannot analyze diploide genetic information

Gene	Species	*n*	no.	*S*	*k*	*h* (±SD)	*π* (±SD)
*COI*	*M. jurtina*	47	20	40	6.29	0.834 ± 0.048	0.01065 ± 0.00185
	*M. nurag*	19	8	33	10.60	0.871 ± 0.044	0.01849 ± 0.00213
	*M. megala*	5	1	–	–	–	–
	*M. cypricola*	12	6	26	8.86	0.848 ± 0.074	0.01497 ± 0.00461
	*M. chia*	16	5	15	2.26	0.65 ± 0.108	0.00474 ± 0.00283
	*M. telmessia*	11	7	30	6.47	0.873 ± 0.089	0.00985 ± 0.00491
	*M. halicarnassus*	8	4	7	2.61	0.750 ± 0.139	0.00397 ± 0.00114
	Lineage A	31	14	17	3.71	0.890 ± 0.036	0.00627 ± 0.00059
	Lineage B	87	23	42	7.27	0.864 ± 0.025	0.01585 ± 0.00138
	All samples	118	37	48	8.84	0.919 ± 0.015	0.01926 ± 0.00099
*CytB*	*M. jurtina*	29	14	30	5.48	0.894 ± 0.040	0.01305 ± 0.00404
	*M. nurag*	12	7	32	11.67	0.909 ± 0.056	0.02804 ± 0.00382
	*M. megala*	5	1	–	–	–	–
	*M. cypricola*	14	8	25	7.82	0.868 ± 0.068	0.01867 ± 0.00563
	*M. chia*	7	4	24	8.48	0.714 ± 0.181	0.02173 ± 0.00831
	*M. telmessia*	16	7	24	4.00	0.792 ± 0.089	0.00952 ± 0.00490
	*M. halicarnassus*	8	5	6	1.79	0.786 ± 0.151	0.00413 ± 0.00120
	All samples	91	33	43	10.54	0.906 ± 0.021	0.02703 ± 0.00106
*wgl*	*M. jurtina*	30 (60)	11	8	1.47	0.774 ± 0.041	0.00432 ± 0.00044
	*M. nurag*	12 (24)	13	11	2.57	0.906 ± 0.046	0.00754 ± 0.00095
	*M. megala*	5 (10)	7	8	3.62	0.933 ± 0.062	0.00899 ± 0.00106
	*M. cypricola*	11 (22)	6	7	1.05	0.671 ± 0.077	0.00281 ± 0.00066
	*M. chia*	14 (28)	9	5	1.83	0.831 ± 0.051	0.00537 ± 0.00051
	*M. telmessia*	14 (28)	6	3	1.18	0.741 ± 0.067	0.00348 ± 0.00040
	*M. halicarnassus*	5 (10)	3	4	0.96	0.511 ± 0.164	0.00281 ± 0.00137
	All samples	91 (182)	34	25	2.48	0.904 ± 0.011	0.00731 ± 0.00033
*EF 1α*	*M. jurtina*	28 (56)	25	24	1.85	0.881 ± 0.032	0.00183 ± 0.00021
	*M. nurag*	12 (24)	19	52	9.65	0.975 ± 0.021	0.00952 ± 0.00349
	*M. megala*	3 (6)	2	1	0.33	0.333 ± 0.215	0.00032 ± 0.00021
	*M. cypricola*	9 (18)	7	7	1.87	0.784 ± 0.085	0.00183 ± 0.00035
	*M. chia*	17 (34)	11	10	1.43	0.811 ± 0.052	0.00143 ± 0.00020
	*M. telmessia*	13 (26)	7	9	1.39	0.689 ± 0.088	0.00134 ± 0.00035
	*M. halicarnassus*	5 (10)	4	5	1.16	0.533 ± 0.180	0.00112 ± 0.00046
	All samples	87 (174)	53	66	2.72	0.851 ± 0.021	0.00285 ± 0.00060
mtDNA	*M. jurtina*	29	14	38	8.21	0.899 ± 0.036	0.01389 ± 0.00244
	*M. nurag*	9	5	26	12.61	0.861 ± 0.087	0.01974 ± 0.00250
	*M. cypricola*	12	10	51	17.59	0.955 ± 0.057	0.01740 ± 0.00519
	*M. chia*	6	4	35	12.13	0.867 ± 0.129	0.01159 ± 0.00688
	*M. telmessia*	11	7	54	11.45	0.873 ± 0.089	0.01052 ± 0.00570
	*M. halicarnassus*	8	6	13	4.39	0.893 ± 0.111	0.00403 ± 0.00106
	Lineage A	29	19	29	6.13	0.921 ± 0.041	0.00606 ± 0.00078
	Lineage B	51	17	37	9.63	0.904 ± 0.022	0.01776 ± 0.00153
	All samples	80	30	43	10.81	0.946 ± 0.011	0.01994 ± 0.00095
nDNA	*M. jurtina*	26 (52)	33	31	3.40	0.962 ± 0.015	0.00248 ± 0.00022
	*M. nurag*	11 (22)	21	29	6.41	0.996 ± 0.015	0.00457 ± 0.00029
	*M. megala*	3 (6)	4	8	3.93	0.867 ± 0.129	0.00276 ± 0.00059
	*M. cypricola*	9 (18)	11	13	2.93	0.908 ± 0.051	0.00208 ± 0.00039
	*M. chia*	14 (28)	20	14	3.33	0.974 ± 0.016	0.00245 ± 0.00021
	*M. telmessia*	13 (26)	16	12	2.61	0.902 ± 0.049	0.00186 ± 0.00030
	*M. halicarnassus*	5 (10)	5	9	2.11	0.756 ± 0.130	0.00148 ± 0.00071
	All samples	81 (162)	90	62	4.42	0.976 ± 0.005	0.00339 ± 0.00014

**Figure 1:**
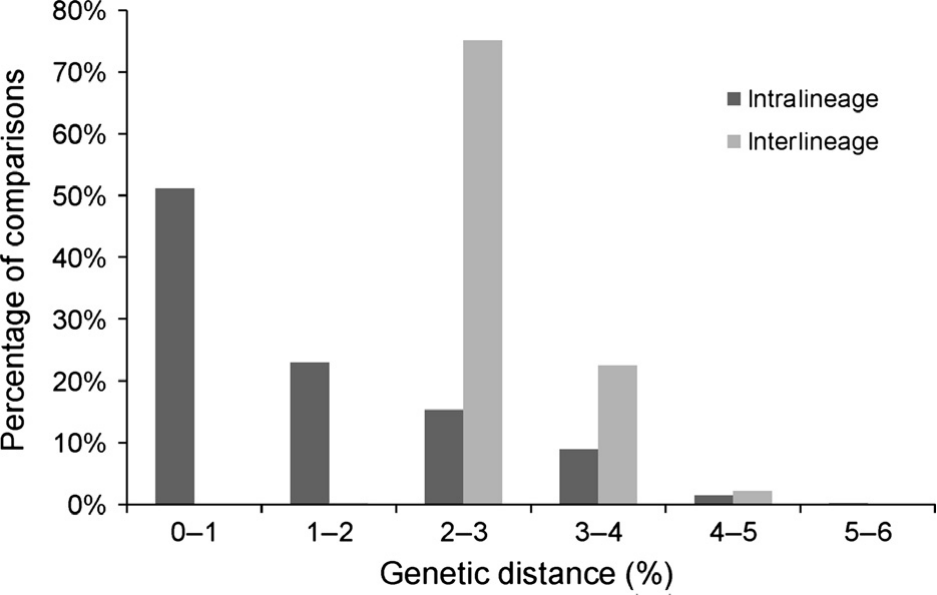
Frequency distribution of pairwise intra- and interlineage Kimura-2-distances of the *COI* sequences. To ensure the usability of DNA barcoding for species delimitation, a “barcoding gap” should exist between these two data series. In *Maniola*, however, genetic distances within lineages versus between lineages intermix.

To use the barcoding region for species identification, a distinct barcoding gap should exist – separating intra- from interspecific pairwise genetic distances. Among the 118 *Maniola COI* sequences, no such gap could be found, as intra- and interspecific distances for conventionally delineated species were intermixing. When only the two major genetic lineages (see below) were compared, genetic distances between and within lineages were separated more clearly from each other, although a convincing barcoding gap could still not be found (Fig.1). Approximately 99.8% of pairwise *COI* sequence comparisons showed two or more percent genetic distance between these two lineages (interlineage), but only 51% of pairwise comparisons showed negligible (0–1% genetic distance) distances within lineages (intralineage). About 26% of sequence comparisons showed high (≥2%) distances within lineages.

### Phylogeny and phylogeography

**Figure 2:**
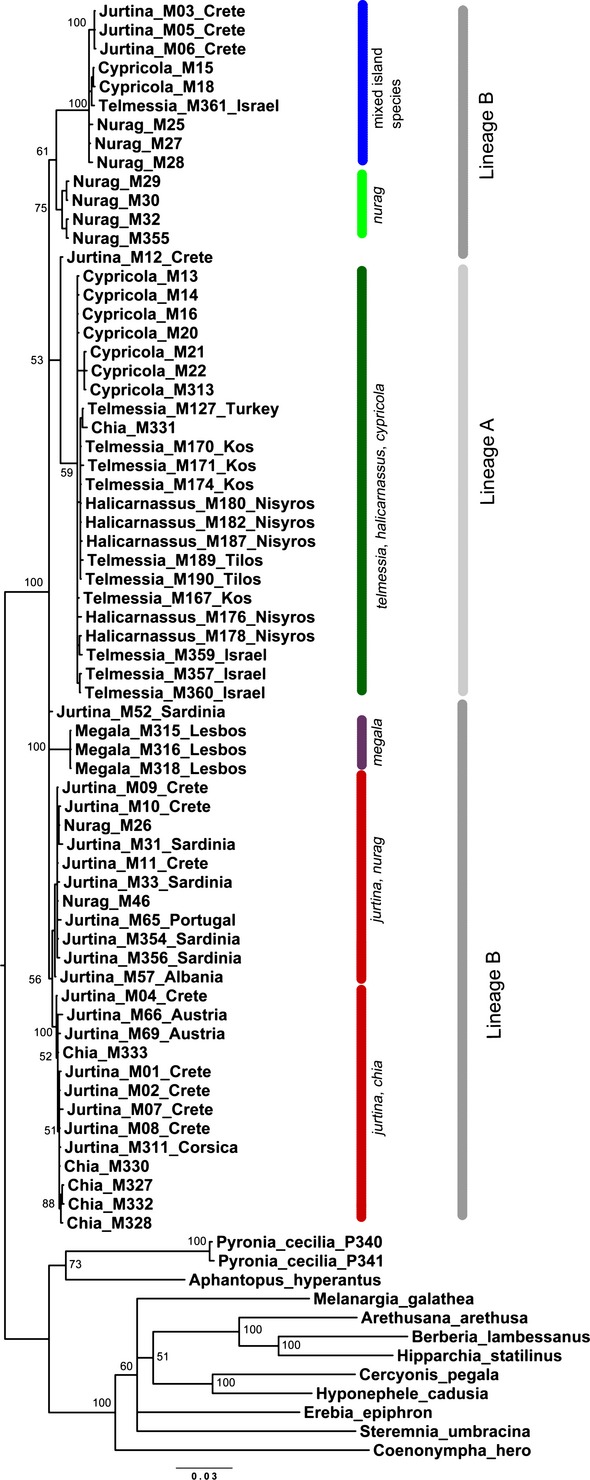
Phylogeny of *Maniola* butterflies, according to Bayesian inference analysis of combined genes dataset with probability values (%). Nominal species do not form clades according to current taxonomy, but two genetic lineages can be roughly defined: one lineage (A) contains *M. telmessia*,*M. halicarnassus,* and *M. cypricola* (59% prob.), and another lineage (B) contains the remaining species. Of the conventionally accepted species, only *M. megala* and *M. halicarnassus* do not occur in several clades across the tree.

**Figure 3:**
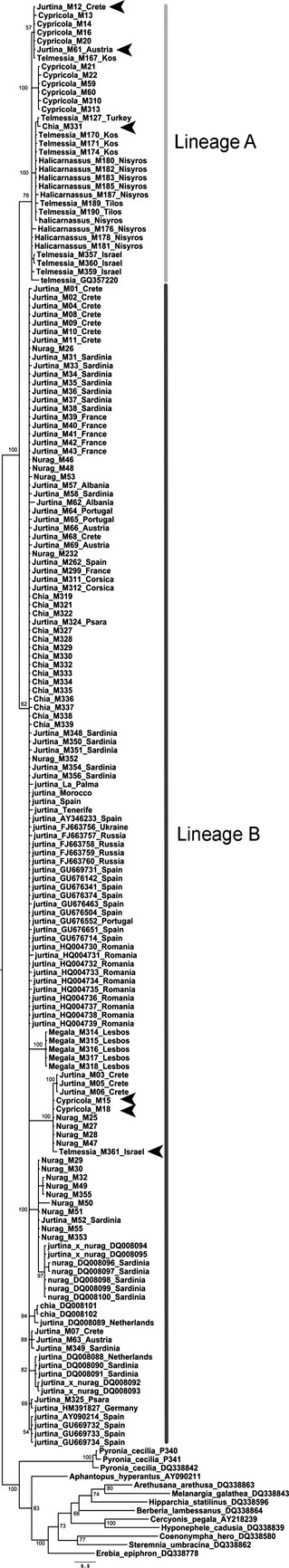
Phylogeny of *Maniola* butterflies, according to Bayesian inference analysis of *COI* sequences with support values. Nominal species form intermixed clades that are not consistent with current taxonomy. Also each of the island endemics spreads through several clades. The only monophylum coinciding with a described species is represented by *M. megala*. The tree shows two main branches (Lineages A and B). Lineage A (light gray) predominantly contains *Maniola telmessia*,*M. halicarnassus,* and *M. cypricola*, whereas (Lineage B; dark gray) contains *M. jurtina*,*M. nurag*,*M. chia,* and *M. megala*. Lineage A shows a probability value of 76%. Black arrows indicate exceptional sequences which cluster with the “wrong” lineage.

The topology of the Bayesian Inference tree of all *Maniola* samples, based on specimens for whom all genetic markers were available plus the selected outgroup species (Fig.2), is not consistent with current taxonomy. Rather, most nominal species form mixed clades. Nevertheless, recurrent clusters can be recognized: there is one lineage of *M. jurtina* (from various provenances; mixed with *M. chia*,*M. nurag,* and *M. megala*) (Lineage B in the lower part of the tree diagram); another one containing a few *M. jurtina* (from Crete), *M. nurag*, two *M. cypricola* specimens, and one *M. telmessia* sequence (upper Lineage B); and one *M. telmessia* (together with all *M. halicarnassus* and the majority of *M. cypricola* sequences) lineage (Lineage A). In the BI tree of COI sequences (Fig.3), Lineage B forms a single clade, so the two parts of this lineage are both called Lineage B in the BI tree of the combined genes, as Lineage B can be defined as all specimens that do not belong to Lineage A. The “mixed island species clade” within Lineage B (marked in blue in Fig.2) contains *M. nurag*,*M. jurtina* from Crete, *M. cypricola,* and a single *M. telmessia* sequence from Israel. All other *M. telmessia* specimens cluster together in one clade (Lineage A). This also applies to *M. halicarnassus*, while *M. megala* forms a distinct clade within Lineage B. Sequences of the island endemics never form coherent clusters. All samples of *M. cypricola* and *M. nurag* distribute across several different clades. Most individuals of *M. chia* cluster with one *M. jurtina* lineage, but one individual appears in the same *M. telmessia*/*M. halicarnassus* cluster that also contains most *M. cypricola* individuals. Overall, the monophyly of *Maniola* is very well supported, whereas most clusters within *Maniola* show low support. Stable, well-supported groupings are only the invariant *M. megala* samples from Lesbos and the aforementioned “mixed island species clade” within Lineage B.

The tree based exclusively on the *COI* barcoding sequences shows similar groupings (Fig.3), but more clearly emphasizes the split of haplotypes into two main lineages: One lineage (Lineage A; light gray) predominantly contains *Maniola telmessia*,*M. halicarnassus,* and *M. cypricola*, whereas the other contains *M. jurtina*,*M. nurag*,*M. chia,* and *M. megala* (Lineage B; dark gray), although both with some exceptions (black arrows). Lineage A shows a probability value of 76%.

**Figure 4:**
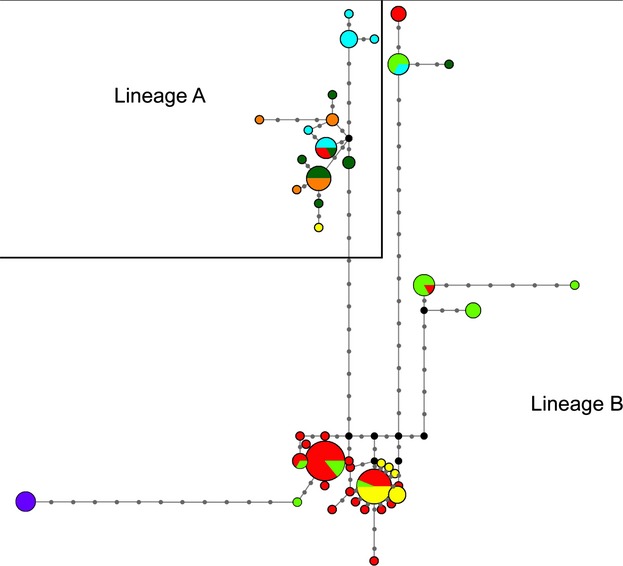
Median-joining network of *COI* sequences of Meadow Brown butterflies (*Maniola*). Size of circles is proportional to number of sequences with similar haplotypes; length of lines is proportional to number of mutational steps between haplotype clades. Black dots are missing haplotypes; gray dots are mutational steps. Five different haplotype clades can be recognized. Species color codes: red = *M. jurtina*, light green *= M. nurag*, yellow = *M. chia*, violet = *M. megala*, blue = *M. cypricola*, dark green = *M. telmessia*, orange = *M. halicarnassus*. Down in the middle two different *Maniola jurtina* clades or lineages can be seen, to the left of them is the distinct *M. megala* clade. To the right side, a *Maniola nurag* clade (with a single *M. jurtina* from Sardinia) can be found. On the top, there is the *M. telmessia*,*M. halicarnassus,* and *M. cypricola* clade to the left and the mixed island species clade (*M. jurtina* from Crete, *M. nurag*,*M. cypricola,* and one *M. telmessia* individual from Israel) to the right. Single haplotypes are common to all clades.

Median-joining networks were calculated for *COI*, mtDNA, nDNA, and combined genes and resembled the phylogenetic trees largely; but in comparison, they show a clearer resolution of the relationships between haplotypes. Networks of *COI*, mtDNA, and combined genes were much alike: they showed the same underlying clustering, but the more genes were used the more single haplotypes and mutational steps between clusters were found. Only the *COI* network is shown (Fig.4), as it is the most clearly arranged. Groupings are similar as in the phylogenetic trees described above.

## Discussion

Our data indeed show one of the – at least up to now – rare cases (cf. Vila et al. 2010), where the differentiation of several nominally described, but rather ambiguously characterized species, did not receive stronger support and higher resolution of “cryptic diversity” by use of genetic methods. Although one might have suspected the existence of even more cryptic species among *Maniola* because of their geographic distribution around the Mediterranean Sea and its many islands, the opposite was found, viz. according to our data, the whole genus consists of only one, quite variable species.

### DNA barcoding, phylogenies, and taxonomic implications

Haplotype as well as nucleotide diversity of the whole *Maniola* genus is comparable with those of a single widely distributed species (e.g., Jeratthitikul et al. 2013). The high genetic diversity of the island species within *Maniola* indicates gene flow among the so-called species. Island populations (Frankham 1997), and isolated populations in general (Cassel and Tammaru 2003), typically have less genetic variation than continental populations due to bottlenecks experienced by small founder populations and subsequent inbreeding (Frankham 1998; Keller and Waller 2002). These outcomes are in accordance with earlier results by Grill et al. (2007), who also found no evidence for lower genetic variation in Sardinian populations of Meadow Brown butterflies.

DNA barcoding based on *COI* sequences cannot be reliably used to split *Maniola* further into a number of species, in contrast to many cases reported in the recent barcoding literature (Hausmann et al. 2011; Ratnasingham and Hebert 2013). The tree constructed from the barcoding region of the *COI* gene shows a clear split into only two lineages, which both range across boundaries of all species taxonomically described for this genus. Addition of further sequence markers did not enhance resolution of clades. All individuals (with the exception of *M. megala*) invariably clustered according to a different pattern than expected from their current taxonomic affiliation.

The results of this study suggest that, instead of accepting seven distinct “good” species, it is more parsimonious to assign all Meadow Browns to one single genetic group. One may distinguish the two lineages A and B as different genetic entities, with some weak further substructure according to haplotypes.

It is understandable that a taxon morphologically as variable as *Maniola* tempted taxonomists of the 19th and 20th century to describe new species; but according to our genetic data, these descriptions delineate variation, not speciation. For example, the species *M. chia* and *M. halicarnassus* (which were proposed as distinct taxa as late as 1987 and 1990, respectively) probably should have been investigated more critically before describing them as new species. If there is endemicity on an island, it is usually observed in a number of unrelated groups of organisms (cf. the high number of endemic species on the Tyrrhenian islands or the Azores). On Chios, as a matter of fact, besides *M. chia,* only the terrestrial isopod *Trachelipus buddelundi* is supposed to be endemic (“Only known from its original description. A doubtful species.”; Alexiou and Sfenthourakis 2013). Within the genus *Maniola*, wing patterns or genitalia morphology are not only determined through the genotype, but also by environmental conditions influencing larval development (Thomson 1973; Goulson 1993; Grill et al. 2004). Hence, even if significant and substantial, differences in phenotypes alone are insufficient to delineate taxonomic entities in that group of butterflies.

Interestingly, a dichotomy – like the two lineages we found – within the “super species” (or species complex) *M. jurtina* has been postulated multiple times. Starting from Tauber (1970), also Hesselbarth et al. (1995) recognized groupings that were assigned to the more western *M. jurtina* sensu stricto on the one hand and the eastern *M. telmessia* on the other hand. Later on, Schmitt et al. (2005) reported the existence of an eastern and a western genetic lineage of *Maniola jurtina* based on allozyme data, wing patterns, and genitalia morphology as did Dapporto et al. (2011) based on genitalia morphology. Dapporto et al. (2011) rightly called *M. jurtina* “enigmatic”, as they found contradictory patterns between allozyme and morphological data. Specimens from continental Italy, Sicily, and North Africa shared the same allozyme set, but their genitalia shape was more similar to specimens from the Balkans and some individuals from Eastern and Central Europe. Their explanation for this discrepancy is “recent gene flow in the wake of postglacial range expansions and shifts” (Dapporto et al. 2011). We hypothesize that these contrasting patterns could have come about through by chance-biased sampling from the genetic lineage A for the allozyme study and from lineage B for the genitalia analyses. At any rate, genetic lineages connected to the main Pleistocene glacial refugia, as postulated by Hewitt (1996, 1999), for example, structured according to the three large Mediterranean peninsulas, could not be detected within *Maniola*.

### Hypothetical evolutionary scenario of the genus *Maniola* and possible expansion routes

Schmitt et al. (2005) hypothesized postglacial expansion routes of an eastern and a western *M. jurtina* lineage, including a hybrid zone, based on allozyme data. Habel et al. (2009) postulated four postglacial recolonization pathways of *Maniola jurtina* (three to northern Europe, one to North Africa) out of the Mediterranean peninsulas, also based on allozyme data. Another possible scenario concerning the origin and expansion routes of the whole genus was much earlier suggested by Tauber (1970), who postulated that the western *M. jurtina* lineage and the eastern *M. telmessia* species complex diverged from North African ancestors. According to Tauber, the *M. jurtina* lineage spreads westward over Gibraltar and the Iberian Peninsula, while the *M. telmessia* complex expanded through Palestine, Lebanon, and Syria to the east. This hypothesis coincides with the fact that *M. telmessia* is the only *Maniola* “species” occurring in Israel, Jordan, Lebanon, and Syria (Tshikolovets 2011). According to Tauber's hypothesis, the western *M. jurtina* complex spreads across Europe and would have met the eastern *M. telmessia* complex in Asia Minor, giving rise to a sympatric occurrence of both lineages in the eastern Mediterranean nowadays.

Although over 40 years old, Tauber's (1970) “out-of-Africa” hypothesis seems to be a likely scenario for the evolution of the genetic diversity we find in *Maniola* today. A number of other recent studies revealed similar scenarios for other butterfly species (Weingartner et al. 2006; Kodandaramaiah and Wahlberg 2009; Habel et al. 2010; Husemann et al. 2014). Another fact fitting to an African origin of *Maniola* is their current distribution: while closely related Satyrinae taxa such as *Hyponephele* sp. and *Aphantopus* sp. show trans-Eurasian distributions, the range of *Maniola jurtina* ends roughly at the Ural Mountains (Tshikolovets 2011).

**Figure 5:**
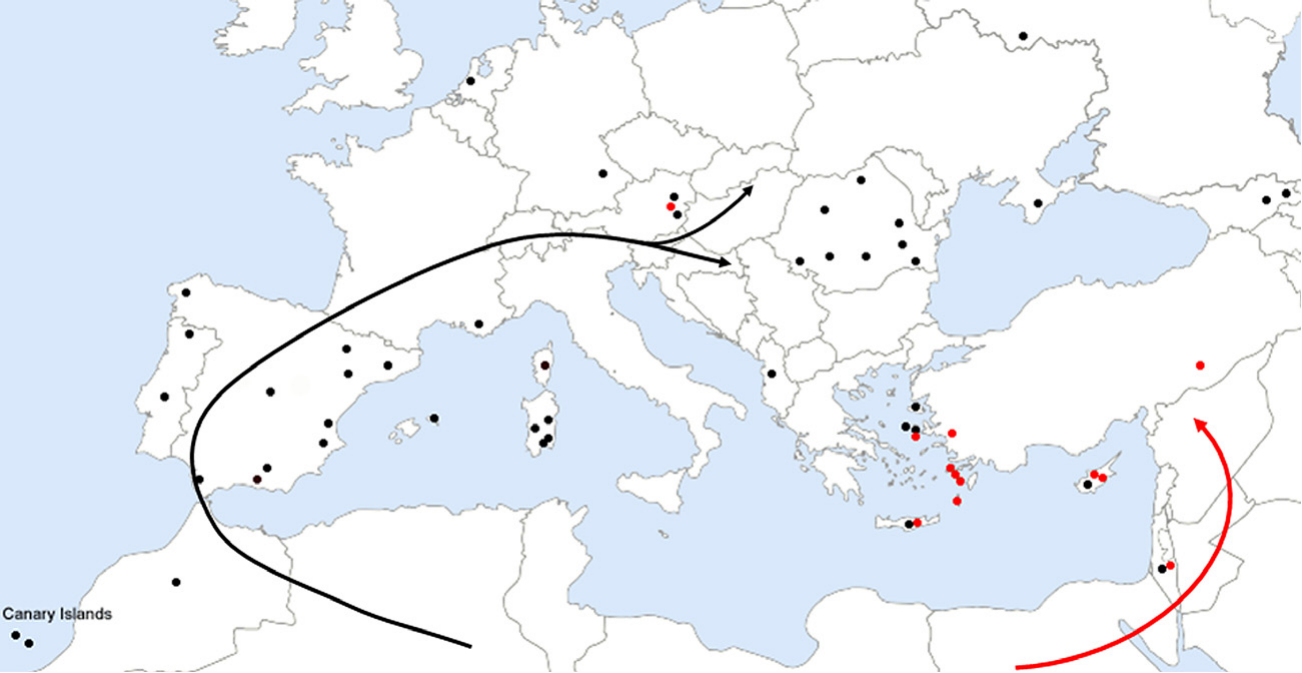
Hypothetical expansion routes of the *Maniola* lineages A (eastern pathway; red) and B (western pathway; black). Dots show collection sites. (Adapted from User: Madman2001/Wikimedia Commons/CC-BY-SA-3.0.).

If the geographic origin of *Maniola* lay in northern Africa (as it is for other nymphalid butterflies: Aduse-Poku et al. 2009; Kodandaramaiah and Wahlberg 2007), the butterflies could have spread northwestward over Gibraltar and the Iberian Peninsula and then eastward all over Europe and into western Asia. A convincing scenario is exactly what Tauber (1970) hypothesized based on morphological characters and paleo-ecological considerations: a split in the North African stem population, with one part dispersing to the west and the other one to the east. Considering our data, Lineage A (*M. cypricola*,*M. telmessia* and *M. halicarnassus*) could have expanded along an eastern pathway, and Lineage B (*M. jurtina*,*M. nurag*,*M. chia,* and *M. megala*) could have colonized western and Central Europe through a western migration route (Fig.5). The few individuals that cluster with the “wrong” lineage might indicate a zone of rather recent intermixture in the Aegean Sea and Central Europe. Of course, to further investigate this hypothesis, more haplotypes from this hybrid zone as well as from northern Africa must be examined.

## Conclusions

During the last decade, phylogenetic studies using sequence data revealed numerous examples for the unexpected discovery of many cryptic species. Our study presents the not so common opposite case: various distinct nominal species melting together to a single species complex. We did not find any proof for the existence of the seven morphologically defined species with the genetic methods used; instead, we found two but moderately distinct genetic lineages. As to the formation of the two lineages, we hypothesize an origin in Africa and two different expansion routes emerging from there, as postulated by Tauber (1970). Thus, we suggest addressing the whole genus as one “super species” *M. jurtina*. Numerous studies in the past few years have strikingly uncovered similar “out-of-Africa” examples, lending further support to this idea for the satyrine genus *Maniola*. Future studies, with a larger set of samples from the African continent, will be needed to evaluate our hypothesis. Most importantly, our results raise the controversial question whether oversplitting of species, despite all the contrasting evidence in recent bar code studies, might be more common than expected until now.

## Supporting Information

**Table S1.**Sequences from GenBank with accession numbers used in this study. All sequences from Peña et al. (2006), except *CytB* sequences. **Table S2**. List of specimens used in this study and their collection localities and dates, when available.
