# Supplementary material for: Tracing the radiation of Maniola (Nymphalidae) butterflies: new insights from phylogeography hint at one single incompletely differentiated species complex
Source: Ecol Evol. 2014 Dec 4;5(1):46–58. doi: 10.1002/ece3.1338 (PMC4298433; doi:10.1002/ece3.1338)
Supplement: Supplementary file 1 — Table S1.Sequences from GenBank with accession numbers used in this study. All sequences from Peña et al. (2006), except CytB sequences. Table S2. List of specimens used in this study and their collection localities and dates, when available. [file ece30005-0046-sd1.docx]

**Supporting Information**

Table S1 Sequences from GenBank with accession numbers used in this study. All sequences from Peña et al. (2006), except *CytB* sequences.

| Species | Locality | Species-ID | *COI* | *EF-1α* | *wgl* | *CytB* |
| --- | --- | --- | --- | --- | --- | --- |
| *Hyponephele cadusia* | Iran | CP10-07 | DQ338839 | DQ338989 | DQ338702 | JQ996403 |
| *Cercyonis pegala* | USA | EW8-2 | AY218239 | AY218259 | AY218277 | - |
| *Aphantopus hyperanthus* | Sweden | EW2-1 | AY090211 | AY090177 | AY090144 | JQ924450 |
| *Erebia epiphron* | France | EW24-3 | DQ338778 | DQ338921 | DQ338638 | EF545676 |
| *Coenonympha hero* | Russia | CP-AC23-26 | DQ338580 | DQ338919 | DQ338636 | JQ924449 |
| *Melanargia galathea* | France | EW24-17 | DQ338843 | DQ338993 | DQ338706 | JQ924453 |
| *Berberia lambessanus* | Morocco | EW26-29 | DQ338864 | DQ339019 | GQ357379 | - |
| *Hipparchia statilinus* | Greece | EW25-24 | DQ338596 | DQ339024 | DQ338733 | - |
| *Arethusana arethusana* | Spain | CP11-06 | DQ338863 | DQ339018 | DQ338728 | - |
| *Steremnia umbracina* | Peru | CP07-89 | DQ338862 | DQ339016 | DQ338726 | - |

Table S2 List of specimens used in this study and their collection localities and dates, when available.

| Specimen code | Species | Region | | Date |
| --- | --- | --- | --- | --- |
| Jurtina_M01_Crete | *Maniola jurtina* | Crete | | 2009 |
| Jurtina_M02_Crete | *Maniola jurtina* | Crete | | 2009 |
| Jurtina_M03_Crete | *Maniola jurtina* | Crete | | 2009 |
| Jurtina_M04_Crete | *Maniola jurtina* | Crete | | 2009 |
| Jurtina_M05_Crete | *Maniola jurtina* | Crete | | 2009 |
| Jurtina_M06_Crete | *Maniola jurtina* | Crete | | 2009 |
| Jurtina_M07_Crete | *Maniola jurtina* | Crete | | 2009 |
| Jurtina_M08_Crete | *Maniola jurtina* | Crete | | 2009 |
| Jurtina_M09_Crete | *Maniola jurtina* | Crete | | 2009 |
| Jurtina_M10_Crete | *Maniola jurtina* | Crete | | 2009 |
| Jurtina_M11_Crete | *Maniola jurtina* | Crete | | 2009 |
| Jurtina_M12_Crete | *Maniola jurtina* | Crete | | 2009 |
| Cypricola_M13 | *Maniola cypricola* | Cyprus | | 2011 |
| Cypricola_M14 | *Maniola cypricola* | Cyprus | | 2011 |
| Cypricola_M15 | *Maniola cypricola* | Cyprus | | 2011 |
| Cypricola_M16 | *Maniola cypricola* | Cyprus | | 2011 |
| Cypricola_M18 | *Maniola cypricola* | Cyprus | | 2011 |
| Cypricola_M20 | *Maniola cypricola* | Cyprus | | 2011 |
| Cypricola_M21 | *Maniola cypricola* | Cyprus | | 2011 |
| Cypricola_M22 | *Maniola cypricola* | Cyprus | | 2011 |
| Nurag_M25 | *Maniola nurag* | Sardinia | | 2001 |
| Nurag_M26 | *Maniola nurag* | Sardinia | | 2001 |
| Nurag_M27 | *Maniola nurag* | Sardinia | | 2001 |
| Nurag_M28 | *Maniola nurag* | Sardinia | | 2001 |
| Nurag_M29 | *Maniola nurag* | Sardinia | | 2001 |
| Nurag_M30 | *Maniola nurag* | Sardinia | | 2001 |
| Jurtina_M31_Sardinia | *Maniola jurtina* | Sardinia | | 2001 |
| Nurag_M32 | *Maniola nurag* | Sardinia | | 2001 |
| Jurtina_M33_Sardinia | *Maniola jurtina* | Sardinia | | 2001 |
| Jurtina_M34_Sardinia | *Maniola jurtina* | Sardinia | | 2001 |
| Jurtina_M35_Sardinia | *Maniola jurtina* | Sardinia | | 2001 |
| Jurtina_M36_Sardinia | *Maniola jurtina* | Sardinia | | 2001 |
| Jurtina_M37_Sardinia | *Maniola jurtina* | Sardinia | | 2001 |
| Jurtina_M38_Sardinia | *Maniola jurtina* | Sardinia | | 2001 |
| Jurtina_M39_France | *Maniola jurtina* | France | | 2000 |
| Jurtina_M40_France | *Maniola jurtina* | France | | 2000 |
| Jurtina_M41_France | *Maniola jurtina* | France | | 2000 |
| Jurtina_M42_France | *Maniola jurtina* | France | | 2000 |
| Jurtina_M43_France | *Maniola jurtina* | France | | 2000 |
| Jurtina_M45_France | *Maniola jurtina* | France | | 2000 |
| Nurag_M46 | *Maniola nurag* | Sardinia | | 2001 |
| Nurag_M47 | *Maniola nurag* | Sardinia | | 2001 |
| Nurag_M48 | *Maniola nurag* | Sardinia | | 2001 |
| Nurag_M49 | *Maniola nurag* | Sardinia | | 2001 |
| Nurag_M50 | *Maniola nurag* | Sardinia | | 2001 |
| Nurag_M51 | *Maniola nurag* | Sardinia | | 2001 |
| Jurtina_M52_Sardinia | *Maniola jurtina* | Sardinia | | 2001 |
| Nurag_M53 | *Maniola nurag* | Sardinia | | 2001 |
| Nurag_M55 | *Maniola nurag* | Sardinia | | 2001 |
| Jurtina_M57_Albania | *Maniola jurtina* | Albania | | 2011 |
| Jurtina_M58_Sardinia | *Maniola jurtina* | Sardinia | | 2001 |
| Cypricola_M59 | *Maniola cypricola* | Cyprus | | 2011 |
| Cypricola_M60 | *Maniola cypricola* | Cyprus | | 2011 |
| Jurtina_M61_Austria | *Maniola jurtina* | Austria | | 2011 |
| Jurtina_M62_Albania | *Maniola jurtina* | Albania | | 2011 |
| Jurtina_M63_Austria | *Maniola jurtina* | Austria | | 2011 |
| Jurtina_M64_Portugal | *Maniola jurtina* | Portugal | | 2011 |
| Jurtina_M65_Portugal | *Maniola jurtina* | Portugal | | 2011 |
| Jurtina_M66_Austria | *Maniola jurtina* | Austria | | 2011 |
| Jurtina_M68_Crete | *Maniola jurtina* | Crete | | 2009 |
| Jurtina_M69_Austria | *Maniola jurtina* | Austria | | 2011 |
| Megala_M83_Turkey | *Maniola megala* | Turkey | | 01.06.1980 |
| Jurtina_M97_Georgia | *Maniola jurtina* | Georgia | | 29.07.2007 |
| Jurtina_M98_Georgia | *Maniola jurtina* | Georgia | | 30.07.2007 |
| Chia_M117 | *Maniola chia* | Chios, Greece | | 23.05.2006 |
| Telmessia_M124_Turkey | *Maniola telmessia* | Turkey | | 16.05.2008 |
| Telmessia_M127_Turkey | *Maniola telmessia* | Turkey | | 08.05.2011 |
| Telmessia_M128_Turkey | *Maniola telmessia* | Turkey | | 08.05.2011 |
| Telmessia_M167_Kos | *Maniola telmessia* | Kós, Greece | | 02.06.2011 |
| Telmessia_M169_Kos | *Maniola telmessia* | Kós, Greece | | 02.06.2011 |
| Telmessia_M170_Kos | *Maniola telmessia* | Kós, Greece | | 02.06.2011 |
| Telmessia_M171_Kos | *Maniola telmessia* | Kós, Greece | | 03.06.2011 |
| Telmessia_M174_Kos | *Maniola telmessia* | Kós, Greece | | 04.06.2011 |
| Halicarnassus_M176_Nisyros | *Maniola halicarnassus* | Nísyros, Greece | | 05.06.2011 |
| Halicarnassus_M178_Nisyros | *Maniola halicarnassus* | Nísyros, Greece | | 05.06.2011 |
| Halicarnassus_M180_Nisyros | *Maniola halicarnassus* | Nísyros, Greece | | 05.06.2011 |
| Halicarnassus_M181_Nisyros | *Maniola halicarnassus* | Nísyros, Greece | | 05.06.2011 |
| Halicarnassus_M182_Nisyros | *Maniola halicarnassus* | Nísyros, Greece | | 05.06.2011 |
| Halicarnassus_M183_Nisyros | *Maniola halicarnassus* | Nísyros, Greece | | 05.06.2011 |
| Halicarnassus_M185_Nisyros | *Maniola halicarnassus* | Nísyros, Greece | | 05.06.2011 |
| Halicarnassus_M187_Nisyros | *Maniola halicarnassus* | Nísyros, Greece | | 05.06.2011 |
| Telmessia_M189_Tilos | *Maniola telmessia* | Tílos, Greece | | 08.06.2011 |
| Telmessia_M190_Tilos | *Maniola telmessia* | Tílos, Greece | | 08.06.2011 |
| Telmessia_M193_Karpathos | *Maniola telmessia* | Kárpathos, Greece | | 09.06.2011 |
| Telmessia_M199_Karpathos | *Maniola telmessia* | Kárpathos, Greece | | 11.06.2011 |
| Nurag_M202 | *Maniola nurag* | Sardinia | | 09.06.2000 |
| Nurag_M206 | *Maniola nurag* | Sardinia | | 17.06.2000 |
| Nurag_M220 | *Maniola nurag* | Sardinia | | 17.06.2000 |
| Nurag_M232 | *Maniola nurag* | Sardinia | | 13.06.2000 |
| Jurtina_M262_Spain | *Maniola jurtina* | Spain | | 13.05.2000 |
| Jurtina_M299_France | *Maniola jurtina* | France | | 30.05.1999 |
| Cypricola_M310 | *Maniola cypricola* | Cyprus | | 2011 |
| Jurtina_M311_Corsica | *Maniola jurtina* | Corsica | | 23.07.2000 |
| Jurtina_M312_Corsica | *Maniola jurtina* | Corsica | | 23.07.2000 |
| Cypricola_M313 | *Maniola cypricola* | Cyprus | | 2011 |
| Megala_M314_Lesbos | *Maniola megala* | Lesbos, Greece | | 09.06.2012 |
| Megala_M315_Lesbos | *Maniola megala* | Lesbos, Greece | | 07.06.2012 |
| Megala_M316_Lesbos | *Maniola megala* | Lesbos, Greece | | 07.06.2012 |
| Megala_M317_Lesbos | *Maniola megala* | Lesbos, Greece | | 09.06.2012 |
| Megala_M318_Lesbos | *Maniola megala* | Lesbos, Greece | | 07.06.2012 |
| Chia_M319 | *Maniola chia* | Chios, Greece | | 26.05.2012 |
| Chia_M320 | *Maniola chia* | Chios, Greece | | 26.05.2012 |
| Chia_M321 | *Maniola chia* | Chios, Greece | | 26.05.2012 |
| Chia_M322 | *Maniola chia* | Chios, Greece | | 26.05.2012 |
| Jurtina_M324_Psara | *Maniola jurtina* | Psara, Greece | | 29.05.2012 |
| Jurtina_M325_Psara | *Maniola jurtina* | Psara, Greece | | 29.05.2012 |
| Chia_M327 | *Maniola chia* | Chios, Greece | | 09/2002 |
| Chia_M328 | *Maniola chia* | Chios, Greece | | 09/2002 |
| Chia_M329 | *Maniola chia* | Chios, Greece | | 09/2002 |
| Chia_M330 | *Maniola chia* | Chios, Greece | | 09/2002 |
| Chia_M331 | *Maniola chia* | Chios, Greece | | 09/2002 |
| Chia_M332 | *Maniola chia* | Chios, Greece | | 09/2002 |
| Chia_M333 | *Maniola chia* | Chios, Greece | | 09/2002 |
| Chia_M334 | *Maniola chia* | Chios, Greece | | 09/2002 |
| Chia_M335 | *Maniola chia* | Chios, Greece | | 09/2002 |
| Chia_M336 | *Maniola chia* | Chios, Greece | | 09/2002 |
| Chia_M337 | *Maniola chia* | Chios, Greece | | 09/2002 |
| Chia_M338 | *Maniola chia* | Chios, Greece | | 09/2002 |
| Chia_M339 | *Maniola chia* | Chios, Greece | | 09/2002 |
| Pyronia_cecilia_P340 | *Pyronia cecilia* | Italy | | 01.06.2002 |
| Pyronia_cecilia_P341 | *Pyronia cecilia* | Italy | | 01.06.2002 |
| Jurtina_M342_Sardinia | *Maniola jurtina* | Sardinia | | 10.05.2001 |
| Nurag_M343 | *Maniola nurag* | Sardinia | | 04.06.2012 |
| Nurag_M344 | *Maniola nurag* | Sardinia | | 04.06.2012 |
| Nurag_M345 | *Maniola nurag* | Sardinia | | 04.06.2012 |
| Cypricola_M346 | *Maniola cypricola* | Cyprus | | 2011 |
| Cypricola_M347 | *Maniola cypricola* | Cyprus | | 2011 |
| Jurtina_M348_Sardinia | *Maniola jurtina* | Sardinia | | 06/2012 |
| Jurtina_M349_Sardinia | *Maniola jurtina* | Sardinia | | 06/2012 |
| Jurtina_M350_Sardinia | *Maniola jurtina* | Sardinia | | 06/2012 |
| Jurtina_M351_Sardinia | *Maniola jurtina* | Sardinia | | 06/2012 |
| Nurag_M352 | *Maniola nurag* | Sardinia | | 06/2012 |
| Nurag_M353 | *Maniola nurag* | Sardinia | | 06/2012 |
| Jurtina_M354_Sardinia | *Maniola jurtina* | Sardinia | | 09/2013 |
| Nurag_M355 | *Maniola nurag* | Sardinia | | 09/2013 |
| Jurtina_M356_Sardinia | *Maniola jurtina* | Sardinia | | 09/2013 |
| Telmessia_M357_Israel | *Maniola telmessia* | Israel | | 09/2013 |
| Telmessia_M359_Israel | *Maniola telmessia* | Israel | | 09/2013 |
| Telmessia_M360_Israel | *Maniola telmessia* | Israel | | 09/2013 |
| Telmessia_M361_Israel | *Maniola telmessia* | Israel | | 09/2013 |
| chia_DQ008101 | *Maniola chia* | Chios, Greece | | 2002 |
| chia_DQ008102 | *Maniola chia* | Chios, Greece | | 2002 |
| halicarnassus_Nisyros | *Maniola halicarnassus* | Nísyros, Greece | | 03.06.2009 |
| jurtina_La_Palma | *Maniola jurtina* | La Palma | | 1996 |
| jurtina_Morocco | *Maniola jurtina* | Morocco | | - |
| jurtina_Spain | *Maniola jurtina* | Spain | | 20.07.2001 |
| jurtina_Tenerife | *Maniola jurtina* | Tenerife | | 22.07.1990 |
| jurtina_AY090214_Spain | *Maniola jurtina* | Spain | - | |
| jurtina_AY346233_Spain | *Maniola jurtina* | Spain, Galicia | - | |
| jurtina_DQ008088_Netherlands | *Maniola jurtina* | Netherlands, Amsterdam | 2001 | |
| jurtina_DQ008089_Netherlands | *Maniola jurtina* | Netherlands, Amsterdam | 2001 | |
| jurtina_DQ008090_Sardinia | *Maniola jurtina* | Italy, Sardinia | 2001 | |
| jurtina_DQ008091_Sardinia | *Maniola jurtina* | Italy, Sardinia | 2001 | |
| jurtina_FJ663756_Ukraine | *Maniola jurtina* | Ukraine | 20.07.2000 | |
| jurtina_FJ663757_Russia | *Maniola jurtina* | Russia | 26.06.1991 | |
| jurtina_FJ663758_Russia | *Maniola jurtina* | Russia | 26.06.1991 | |
| jurtina_FJ663759_Russia | *Maniola jurtina* | Russia | 26.06.1991 | |
| jurtina_FJ663760_Russia | *Maniola jurtina* | Russia | 26.06.1991 | |
| jurtina_GU669731_Spain | *Maniola jurtina* | Spain, Catalonia | 16.09.2008 | |
| jurtina_GU669732_Spain | *Maniola jurtina* | Spain, Catalonia | 16.09.2008 | |
| jurtina_GU669733_Spain | *Maniola jurtina* | Spain, Catalonia | 02.08.2008 | |
| jurtina_GU669734_Spain | *Maniola jurtina* | Spain, Catalonia | 29.07.2008 | |
| jurtina_GU676142_Spain | *Maniola jurtina* | Spain, Aragon | 06.07.2008 | |
| jurtina_GU676341_Spain | *Maniola jurtina* | Spain, Cadiz | 07.09.2008 | |
| jurtina_GU676374_Spain | *Maniola jurtina* | Spain, Alicante | 22.08.2008 | |
| jurtina_GU676463_Spain | *Maniola jurtina* | Spain, Granada | 03.06.2008 | |
| jurtina_GU676504_Spain | *Maniola jurtina* | Spain, Valencia | 14.06.2008 | |
| jurtina_GU676552_Portugal | *Maniola jurtina* | Portugal | 26.07.2008 | |
| jurtina_GU676651_Spain | *Maniola jurtina* | Spain, Madrid | 05.06.2008 | |
| jurtina_GU676714_Spain | *Maniola jurtina* | Spain, Balears | 02.06.2008 | |
| jurtina_HM391827_Germany | *Maniola jurtina* | Germany, Bavaria | 17.07.1996 | |
| jurtina_HQ004730_Romania | *Maniola jurtina* | Romania | 29.05.2008 | |
| jurtina_HQ004731_Romania | *Maniola jurtina* | Romania | 26.05.2008 | |
| jurtina_HQ004732_Romania | *Maniola jurtina* | Romania | 02.06.2008 | |
| jurtina_HQ004733_Romania | *Maniola jurtina* | Romania | 07.06.2008 | |
| jurtina_HQ004734_Romania | *Maniola jurtina* | Romania | 29.06.2004 | |
| jurtina_HQ004735_Romania | *Maniola jurtina* | Romania | 09.08.2007 | |
| jurtina_HQ004736_Romania | *Maniola jurtina* | Romania | 01.06.2007 | |
| jurtina_HQ004737_Romania | *Maniola jurtina* | Romania | 06.06.2007 | |
| jurtina_HQ004738_Romania | *Maniola jurtina* | Romania | 22.06.2006 | |
| jurtina_HQ004739_Romania | *Maniola jurtina* | Romania | 25.06.2006 | |
| jurtina_x_nurag_DQ008092 | *Maniola jur x nur* | Sardinia | 2001 | |
| jurtina_x_nurag_DQ008093 | *Maniola jur x nur* | Sardinia | 2001 | |
| jurtina_x_nurag_DQ008094 | *Maniola jur x nur* | Sardinia | 2001 | |
| jurtina_x_nurag_DQ008095 | *Maniola jur x nur* | Sardinia | 2001 | |
| nurag_DQ008096_Sardinia | *Maniola nurag* | Sardinia | 2001 | |
| nurag_DQ008097_Sardinia | *Maniola nurag* | Sardinia | 2001 | |
| nurag_DQ008098_Sardinia | *Maniola nurag* | Sardinia | 2001 | |
| nurag_DQ008099_Sardinia | *Maniola nurag* | Sardinia | 2001 | |
| nurag_DQ008100_Sardinia | *Maniola nurag* | Sardinia | 2001 | |
| telmessia_GQ357220 | *Maniola telmessia* | - | - | |
| Pyronia_cecilia_DQ338842 | *Pyronia cecilia* | Spain: Sant Climent | - | |
